# From Broadband to Single-Tone Stimulation: Frequency-Selective Response of *Staphylococcus aureus* Biofilms to Electric Fields

**DOI:** 10.3390/microorganisms14071516

**Published:** 2026-07-11

**Authors:** Marco Balato, Emanuela Roscetto, Maria Rosaria Catania, Martina Aversa, Carlo Petrarca, Massimo Vitelli, Umberto Galdiero, Luigi Costanzo, Valeria Nocerino, Giovanni Balato

**Affiliations:** 1 Department of Electrical Engineering and Information Technologies (DIETI), University of Naples Federico II, Via Claudio 21, 80125 Napoli, Italy; marco.balato@unina.it (M.B.); martina.aversa@unina.it (M.A.); carlo.petrarca@unina.it (C.P.); valeria.nocerino@unina.it (V.N.); 2 Department of Molecular Medicine and Medical Biotechnologies, University of Naples Federico II, Via Pansini 5, 80131 Napoli, Italy; umberto.galdiero@unina.it; 3 Department of Engineering, Università degli Studi della Campania “Luigi Vanvitelli”, Via Roma, 81031 Aversa, Italy; massimo.vitelli@unicampania.it (M.V.); luigi.costanzo@unicampania.it (L.C.); 4 Department of Public Health, University of Naples Federico II, Via Pansini 5, 80131 Napoli, Italy; giovanni.balato@unina.it

**Keywords:** biofilm, *Staphylococcus aureus*, electric field, frequency-dependent response, bioelectric effect

## Abstract

Biofilm-associated infections caused by *Staphylococcus aureus* are highly resistant to antimicrobial treatments. Low-intensity electric fields have shown promise as an antibiofilm strategy; however, the role of frequency remains poorly understood. In this study, mature *S. aureus* biofilms were exposed to low-intensity alternating electric fields (12.5 mV/cm) across broadband (10 Hz–10 MHz) frequency range and band-limited sub-ranges, as well as to selected single-tone frequencies within the interval 1–100 kHz. Antibiofilm activity was assessed in terms of cell culturability (CFU/mL) and biofilm biomass. A significant frequency-dependent effect was observed (*p* < 0.001), with maximal activity observed only at specific frequencies within the range 1–100 kHz, whereas other frequencies were ineffective. These findings demonstrate that the electrical antibiofilm effect is characterized by a frequency selective response, which is similar to a band-pass filter with a nearly flat shape inside the band-pass range and strongly attenuated effects outside such a band. This frequency-selective response supports the hypothesis of a biophysical mechanism involving membrane and matrix interactions and highlights the potential for developing targeted, frequency-optimized electroceutical strategies.

## 1. Introduction

Biofilm-associated infections pose a significant clinical challenge due to their inherent resistance to antimicrobial treatments, accounting for a substantial proportion of chronic and device-related infections and contributing to high rates of treatment failure and recurrence [1,2,3]. Within biofilms, bacterial cells are embedded in a self-produced Extracellular Polymeric Substance (EPS) matrix that limits antibiotic penetration, alters metabolic activity, and promotes persistent infections [4,5,6,7]. These features are of relevance in the context of *Staphylococcus aureus*, a leading pathogen in chronic and device-associated infections, including periprosthetic joint infections, where treatment failure and recurrence are prevalent [1,2,3,8]. In recent years, the application of low-intensity electric fields has emerged as a promising adjunctive strategy for the control of bacterial biofilms. Electrical stimulation has been demonstrated to modulate biofilm structure, increase membrane permeability, and enhance the activity of antimicrobial agents through the so-called “bioelectric effect” [9,10,11,12]. Despite the mounting evidence supporting this approach, the underlying mechanisms governing the interaction between electric fields and biofilms remain only partially understood. Among the various parameters of electrical stimulation, frequency is likely to play a key role, as it may influence charge distribution, membrane polarization, and electrochemical interactions within the biofilm matrix. However, its contribution to the antibiofilm effect remains poorly investigated. Our previous studies demonstrated that mature *S. aureus* biofilms exhibit measurable bioelectrical responses to low-intensity alternating electric fields and identified a transient Electric Field-Induced Vulnerability Window (EFIVW), during which biofilm-associated bacteria become more susceptible to antimicrobial treatment [11,12]. These outcomes suggest that electric fields primarily modulate the structural and physicochemical properties of the biofilm rather than directly altering bacterial susceptibility. Overall, the results support that the interaction between electric fields and biofilms is governed by intrinsic biophysical mechanisms. However, a critical question remains unresolved: whether the antibiofilm effect observed under broadband electrical stimulation is uniformly distributed across the frequency spectrum or is instead driven by specific discrete frequency components. In other words, is the electrical antibiofilm effect inherently broadband in nature or is it spectrally sparse? Addressing this question is essential for both mechanistic understanding and practical optimization of electroceutical strategies. If only a limited number of frequencies contribute to the antibiofilm effect, targeted stimulation protocols could be developed to maximize efficacy while reducing exposure time and energy consumption.

## 2. Materials and Methods

### 2.1. Experimental Setup

An integrated platform was used to expose mature biofilms to low-intensity sinusoidal electric fields, as previously described [11,12]. The system consisted of a cuvette-based exposure chamber, a programmable voltage source, and a dedicated control unit. Biofilms were cultivated on 500 µm-thick Polyethylene Terephthalate (PET) substrates, arranged vertically between two parallel electrodes separated by 0.4 cm. This configuration was employed to ensure the generation of a spatially uniform electric field. A sinusoidal signal was applied across the electrodes, thereby generating an electric field with a Root-Mean-Square (RMS) amplitude of 12.5 mV/cm. The system permitted modulation of excitation frequencies between 10 Hz and 10 MHz. Further technical details of the platform and the signal generation protocol are provided in [11,12].

### 2.2. Bacterial Strain and Growth Conditions

The present study was conducted using the MRSA reference strain ATCC 43300. The strain was cultivated in Brain–Heart Infusion (BHI) broth (Becton Dickinson Diagnostic Systems, Sparks, MD, USA) at 37 °C for 24 h. Aliquots were stored at 80 °C in BHI supplemented with 15% glycerol until further use. The strain’s identification and antibiotic susceptibility profile were determined using the automated BD Phoenix system (Becton Dickinson, Franklin Lakes, NJ, USA).

### 2.3. Biofilm Formation Assay on PET Slides

*Staphylococcus aureus* biofilm was grown for 5 days on sterile polyethylene terephthalate (PET) slides (10 mm × 30 mm × 0.5 mm). First, a bacterial suspension was adjusted to 0.5 McFarland standard and diluted 1:100 in Brain Heart Infusion (BHI) broth supplemented with 1% glucose. The PET slides were immersed in this bacterial suspension and incubated at 37 °C under static aerobic conditions. Fresh BHI supplemented with 1% glucose was replaced every 24 h.

### 2.4. Electrical Exposure Procedure

The Electrical Exposure Procedure (EEP) was performed using a logarithmic frequency sweep (10 Hz–10 MHz) with 10 steps per decade. Specifically, the sweep covered six decades, resulting in 60 discrete frequency steps, with each frequency applied for two seconds during each sweep cycle. One complete sweep lasted for a duration of two minutes. The Electrical Exposure Time (EET) was set at 6 min, corresponding to three consecutive sweep cycles, to ensure comparable experimental conditions. This approach was selected to maintain the same exposure time for each individual frequency as opposed to the same number of sweep cycles across the different experimental conditions. This methodological decision was made in order to enable a more reliable comparison of the relative contribution of each frequency to the overall antibiofilm effect. The applied electrical stimulus corresponded to an RMS electric field of 12.5 mV/cm, obtained by applying an AC voltage of 5 mV across two electrodes separated by 0.4 cm. The selected voltage level was chosen to ensure that the resulting currents remained within the microcurrent regime and well below the safety limits reported for medical electrical devices. This voltage level also enabled the investigation of biofilm responses to low-intensity electrical stimulation. Across the investigated frequencies, the measured current ranged approximately from 1 µA to 35 µA, corresponding to current densities between 0.5 µA/cm^2^ and 17.5 µA/cm^2^. The range of measured values falls within the microcurrent regime [12]. The experimental study was conducted using cuvettes containing 1 mL of 0.9% NaCl solution. Each cuvette contained a single PET slide bearing a mature *Staphylococcus aureus* biofilm, with a total culture age of 96 h. Following electrical exposure, both treated and untreated slides were removed from the cuvettes and gently washed with Phosphate-Buffered Saline (PBS). Each experiment was performed in triplicate. An investigation was conducted into different frequency conditions. In addition to the Full Frequency Range (FFR) (10 Hz–10 MHz), the study encompassed three distinct frequency sub-ranges: SR1 (10 Hz–1 kHz), SR2 (1 kHz–100 kHz), and SR3 (100 kHz–10 MHz) (Table 1). In the sub-range experiments, the logarithmic sweep mode with 10 frequency steps per decade was also implemented. Furthermore, a targeted analysis was performed by testing five individual frequencies within SR2 (Single Frequencies, SFs). The following single frequencies were selected: SF1 = 1.62 kHz, SF2 = 3.36 kHz, SF3 = 8.86 kHz, SF4 = 14.39 kHz, and SF5 = 61.6 kHz (Table 1). For all experimental conditions (full frequency range, sub-ranges, and single-frequency tests), the electrical exposure time and the field amplitude were kept constant (EET = 6 min and 12.5 mV/cm, respectively). Untreated slides were utilized as positive controls. Following the electrical exposure procedure, the residual biofilm activity on the slides was quantitatively assessed in terms of cell culturability (CFU/mL) and biomass (crystal violet) assays.

### 2.5. Colony-Forming Unit (CFU) Assay

The efficacy of the electrical treatment on *S. aureus* biofilms was evaluated by quantifying the bacterial load attributable solely to cultivable cells, expressed as log_10_ (CFU/mL). Subsequently, both the control and test slides were meticulously washed with Phosphate-Buffered Saline (PBS) to remove non-adherent cells. The remaining biofilms were detached and suspended by vortexing for 5 min in a 0.1% dithiothreitol (DTT) solution [13]. Tenfold serial dilutions of the obtained suspensions were plated on Mueller–Hinton agar. After 18–20 h at 37 °C, the resulting colonies were counted. The CFU/mL values were expressed in exponential notation and as the percentage of cell death relative to the untreated control. To ensure complete removal of any residual attached cells, the slides recovered after the DTT treatment were incubated in BHI overnight at 37 °C. Each experiment was performed in triplicate and repeated twice independently.

### 2.6. Crystal Violet Assay

The total biomass of the biofilm was analyzed using the Crystal Violet (CV) staining method. After the electrical exposure procedure, treated and untreated slides were first washed with PBS to remove non-adherent cells and dried for 45 min at 60 °C. The biofilms were stained by incubation for 20 min with 1% crystal violet solution. After incubation, excess crystal violet was removed by washing with PBS, and all slides were then treated with absolute ethanol to elute the dye from the biofilms. Absorbance at 595 nm was measured using a microplate reader (Bio-Rad Laboratories Srl, Hercules, CA, USA) and correlated with the amount of biofilm present. The ratios of treated biofilms were normalized to the untreated control, which was set to 100%.

### 2.7. Statistical Analysis

The experimental data are presented as mean ± standard deviations. The activity of each treatment was evaluated through a dual approach: in terms of CFU reduction (log_10_ scale), and in terms of biomass reduction. Each analysis was performed independently, as the experimental designs did not include crossed factors. Statistical differences among groups were assessed using one-way analysis of variance (ANOVA), followed by Tukey’s post hoc test. Given that each dataset incorporated a single independent variable, designated as the “frequency variable”, the ANOVA was deemed the most suitable statistical approach. Concurrently, comparative analyses were conducted with the Untreated Control (CT) group utilizing the *t*-test. Statistical significance was set at *p* < 0.05. All analyses were conducted using IBM SPSS Statistics for Windows, Version 23.0 (IBM Corp., Armonk, NY, USA).

## 3. Results

### 3.1. Broadband Frequency Response of Biofilm-Impact on Biofilm Cell Culturability

A strong frequency-dependent effect on cell culturability (CFU/mL) was observed following electrical stimulation (*p* < 0.001). The antibiofilm response was confined to the 1–100 kHz range, with no significant effects detected at lower or higher frequencies. A highly significant difference was observed among the treatment groups (*p* < 0.001). The full frequency range (FFR) and the SR2 condition (1–100 kHz) led to a substantial decrease in CFU/mL compared to the untreated control (CT) (7.56 ± 0.05 log_10_ CFU/mL) vs. 8.56 ± 0.18 log_10_ (CFU/mL) and 7.52 ± 0.11 log_10_ (CFU/mL) vs. 8.56 ± 0.18 log_10_ (CFU/mL), respectively; *p* < 0.01). Conversely, SR1 (10 Hz–1 kHz) and SR3 (100 kHz–10 MHz) did not show a statistically significant difference from the control group (*p* > 0.05). No statistically significant difference was observed between the FFR and SR2 conditions. Conversely, both FFR and SR2 showed significantly higher activity compared to SR1 and SR3 (*p* < 0.01). Pairwise comparisons indicated an approximate 1-log_10_ (~90%) reduction in culturable cells for FFR and SR2 compared to the control. These results demonstrate that the antibiofilm effect is localized within a specific frequency band rather than uniformly distributed across the spectrum. The results are summarized in Table 2, and are consistent with previous findings [11], in which mature *S. aureus* biofilms exposed to wide-spectrum electrical stimulation exhibited a pronounced frequency-dependent response, with the most relevant antibiofilm effects observed within the intermediate frequency region (1–100 kHz).

### 3.2. Broadband Frequency Response of Biofilm-Impact on the Biofilm Biomass

To confirm these findings, biofilm biomass was quantified using the crystal violet (CV) assay. Consistent with the CFU results, a highly significant difference among groups was observed (*p* < 0.001). Both FFR and SR2 exhibited a substantial reduction in residual biomass, with mean values of 41.64% ± 2.27 and 40.43% ± 1.07, respectively. Conversely, SR1 and SR3 showed significantly higher biomass levels (91.10% ± 1.91 and 88.03% ± 1.76), comparable to the untreated conditions. Tukey’s post hoc test confirmed that FFR and SR2 differed significantly from SR1 and SR3 (*p* < 0.001), whereas no significant differences were observed between FFR and SR2 or between SR1 and SR3. Pairwise comparisons with the control group further confirmed a significant reduction in biomass for FFR and SR2 (*p* < 0.05), while no significant differences were detected for SR1 and SR3. Collectively, these data indicate that the antibacterial effect of electrical stimulation extends beyond cell viability and involves a biofilm detachment, predominantly within the 1–100 kHz frequency range. The results are illustrated in Table 3 and are consistent with those previously reported in [11].

### 3.3. Single-Tone Frequency Response of Biofilm Impact on Biofilm Cell Culturability

**Figure 1:**
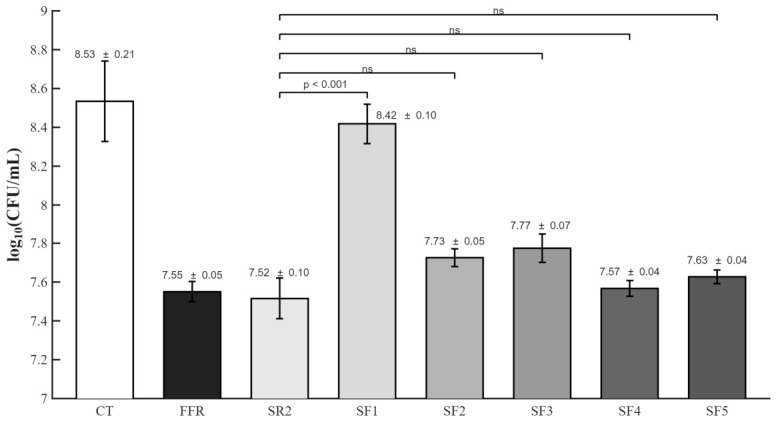
Colony-forming units (CFU/mL) in mature *Staphylococcus aureus* biofilms following single-tone electrical stimulation at different frequencies. Bars represent Mean ± SD (*n* = 3) of log_10_ (CFU/mL). Treatments included five discrete frequencies within the SR2 range: SF1 (1.62 kHz), SF2 (3.36 kHz), SF3 (8.86 kHz), SF4 (14.39 kHz), and SF5 (61.6 kHz), along with CT, FFR and the corresponding band-limited condition (SR2). ns = not significant.

To further investigate whether the band-limited effect arises from the entire frequency range or from specific components, single-frequency experiments were performed within the SR2 interval. Single-frequency stimulation revealed a non-uniform response within the SR2 band, indicating that the antibiofilm effect is driven by discrete frequencies rather than by the entire spectrum. The effect of single-tone electrical stimulation on biofilm cell culturability was evaluated by comparing individual frequencies within the SR2 range with the corresponding band-limited condition. A highly significant difference among groups was observed (*p* < 0.001), indicating a strong frequency-dependent response. Among the tested conditions, SF1 (1.62 kHz) exhibited the highest CFU/mL values (8.43 ± 0.09 log_10_ (CFU/mL)), comparable to untreated conditions (Table 2) and significantly higher than all other frequencies (*p* < 0.001), indicating negligible antibacterial activity. In contrast, SF4 (14.39 kHz) and SF5 (61.6 kHz) showed the lowest CFU levels (7.57 ± 0.04 log_10_ (CFU/mL) and 7.63 ± 0.04 log_10_ (CFU/mL), respectively), comparable to the SR2 band-limited condition (7.52 ± 0.11 log_10_ (CFU/mL)), with no significant differences among these groups. SF2 (3.36 kHz) and SF3 (8.86 kHz) showed numerically intermediate CFU values (7.73 ± 0.05 log_10_ (CFU/mL) and 7.78 ± 0.07 log_10_ (CFU/mL), respectively); however, they did not differ significantly from SR2, SF4, or SF5 (*p* > 0.05). These results further demonstrate that the antibiofilm activity is confined to the SR2 frequency band, within which multiple frequencies are capable of producing comparable biological effects. SF1 (1.62 kHz) showed CFU levels comparable to the untreated control, indicating a complete lack of antibacterial activity; in contrast, SF4 (14.39 kHz) and SF5 (61.6 kHz) reproduced the full band-limited effect, with CFU levels comparable to SR2. These results demonstrate that the antibiofilm effect is localized within a specific frequency band rather than uniformly distributed across the spectrum (Figure 1).

### 3.4. Single-Tone Frequency Response of Biofilm Impact on Biofilm Biomass

**Figure 2:**
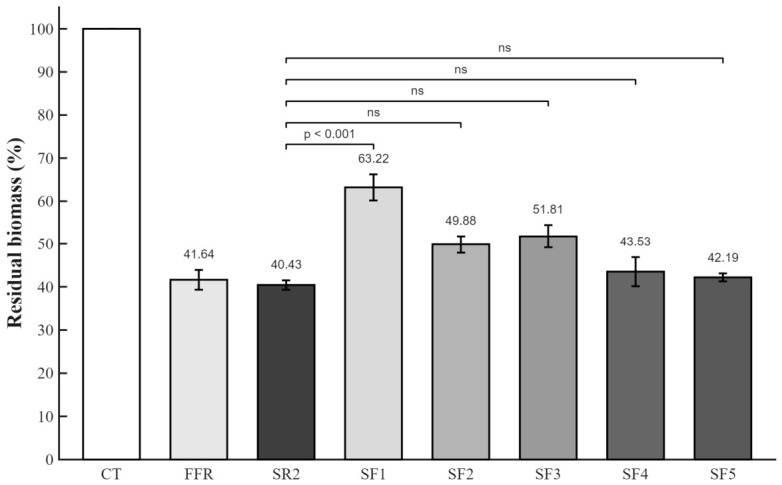
Residual biofilm biomass (%) in mature *Staphylococcus aureus* biofilms following single-tone electrical stimulation at different frequencies. Bars represent Mean ± SD (*n* = 3) of residual biomass quantified by the crystal violet assay. Treatments included five discrete frequencies within the SR2 range: SF1 (1.62 kHz), SF2 (3.36 kHz), SF3 (8.86 kHz), SF4 (14.39 kHz), and SF5 (61.6 kHz), along with CT, FFR and the corresponding band-limited condition (SR2). ns = not significant.

The frequency-selective behavior observed in CFU measurements was confirmed at the structural level by biomass analysis (*p* < 0.001). Only selected frequencies (SF4 and SF5) produced biomass reductions comparable to SR2, whereas others showed limited or partial effects. Among the tested conditions, SF1 (1.62 kHz) exhibited the highest residual biomass (63.22% ± 3.00), indicating limited disruption of the biofilm. In contrast, SR2 (40.43% ± 1.07), SF4 (43.53% ± 3.35), and SF5 (42.19% ± 0.93) showed the lowest biomass levels, with no significant differences among these conditions, indicating comparable antibiofilm activity. Intermediate values were observed for SF2 (49.88% ± 1.94) and SF3 (51.81% ± 2.61), suggesting a partial reduction in biofilm biomass. These results are consistent with the CFU results, indicating that the antibacterial effect of electrical stimulation is governed by discrete frequency-dependent mechanisms, associated not only with reduced bacterial viability but also with biofilm biomass loss. Overall, the results demonstrate that the antibiofilm response is driven by specific discrete frequencies and is not a broadband phenomenon (Figure 2).

## 4. Discussion

The present study offers novel insights into the role of frequency in electric field–mediated antibiofilm activity, demonstrating that the observed antibiofilm effect is strongly frequency-dependent and, importantly, not uniformly distributed across the investigated spectrum. Rather than reflecting a broadband phenomenon, the observed response appears to be governed by a limited set of effective frequencies. The results of both colony-forming unit counts and crystal violet biomass measurements consistently demonstrated that the most pronounced antibiofilm effect occurred within the SR2 range (1–100 kHz). In contrast, the lower SR1 and higher SR3 frequency ranges exhibited negligible activity.

A key finding of this study is that the antibiofilm effect observed under broadband stimulation within the SR2 range can be reproduced by selected single-tone frequencies. This indicates that the broadband response does not arise from the cumulative contribution of all spectral components; instead, the antibiofilm activity is confined to a specific frequency band, within which several frequencies are capable of reproducing the observed effect. Specifically, SF4 (14.39 kHz) and SF5 (61.6 kHz) demonstrated antibacterial efficacy that was comparable to that observed with the full SR2 band-limited condition, while other frequencies within the same range (e.g., SF1) exhibited significantly reduced effectiveness. These findings suggest that the biological response of bacterial biofilms to electric field stimulation is strongly frequency-dependent.

Although the present experimental design does not permit a formal assessment of additive or synergistic interactions between frequencies, the equivalence between SR2 and selected single-tone responses supports a frequency-selective mechanism.

The frequency selective response observed in this study can be interpreted in the context of biofilm biophysics. It has been shown in prior studies that bacterial biofilms possess intrinsic electrical properties and respond to external electric stimuli in a frequency-dependent manner [11,12]. Within this framework, the biofilm can be conceptualized as a complex heterogeneous system composed of cells embedded within an extracellular matrix, whose electrical and mechanical properties are closely intertwined. The observed dependence on specific frequency ranges may suggest the presence of characteristic response times or relaxation processes within the biofilm structure, although these mechanisms were not directly investigated in the present study. One possible interpretation is that this behavior is analogous to frequency-dependent responses observed in viscoelastic systems, wherein the mechanical properties of materials are governed by characteristic time constants and specific excitation frequencies [14,15,16,17]. In viscoelastic materials, external stimuli at specific frequencies can induce maximal deformation or energy dissipation, while other frequencies yield minimal effects [18]. Accordingly, the antibiofilm activity observed in the present study could reflect resonance-like interactions between the applied electric field and the structural components of the biofilm. At present, however, this interpretation remains hypothetical and requires direct experimental verification. This interpretation should be regarded as a working hypothesis requiring further experimental validation. From a mechanistic perspective, such interactions could potentially involve frequency-dependent polarization of bacterial membranes, modulation of ion transport, or mechanical perturbation of the extracellular polymeric substance (EPS) matrix.

Such processes could lead to transient destabilization of the biofilm structure and increased susceptibility of embedded bacteria. This interpretation is consistent with our previous findings, which demonstrated that electric field exposure induces structural alterations in mature biofilms and can lead to significant reductions in viability depending on exposure conditions. In fact, in our previous research, a destructive interaction between the applied electric field and the mature *S. aureus* biofilm was observed, making it impossible to accurately measure the frequency response through impedance spectroscopy and thus limiting the assessment of its intrinsic bioelectrical properties [11].

It is noteworthy that the present results can be integrated with our previous research on the Electric Field-Induced Vulnerability Window (EFIVW), in which a transient and reversible phase of increased antibiotic susceptibility was identified following electrical exposure [12]. In the aforementioned study, the EFIVW was delineated as the maximum time interval between the cessation of electrical stimulation and the subsequent administration of the antibiotic, during which enhanced biofilm susceptibility could still be observed under an asynchronous bioelectric approach. Consequently, the transient effects associated with the EFIVW were generated by the interaction between the electric field and the biofilm itself, independently of the simultaneous presence of antibiotics.

In this framework, the present findings suggest that frequency may represent a key control parameter governing the induction of this transient vulnerable state. Specifically, only certain effective frequencies may be involved in triggering the structural and physicochemical perturbations underlying the EFIVW, while others remain biologically ineffective. As indicated by prior research, the bioelectric effect is mechanistically distinct from reverse resistance in planktonic cells. This finding suggests that the primary target of the electric field is not the bacterial cell itself, but rather the biofilm structure [12]. Taken together, these observations are consistent with the hypothesis that electric fields may act by modulating biofilm structural integrity through membrane-level and matrix-level mechanisms. This perspective provides a possible framework for interpreting the relationship between frequency selectivity and the temporal dynamics of biofilm susceptibility. Further mechanistic studies will be required to verify the biophysical processes proposed here.

Nevertheless, some limitations of the present study should be acknowledged. The analysis was restricted to a limited number of discrete frequencies within the SR2 range and further studies are necessary to define the frequency-response profile of biofilms more precisely. In addition, the experimental design did not incorporate controlled combinations of multiple frequencies, which would be necessary to formally assess potential additive or synergistic effects. Furthermore, although the adopted experimental setup was designed to ensure a spatially uniform electric field at the system level, local field inhomogeneities within the heterogeneous three-dimensional biofilm microenvironment cannot be entirely excluded. Consequently, the present configuration does not permit a rigorous assessment of localized resonance phenomena within the biofilm structure.

A further limitation of the present study is that all experiments were conducted using a single reference MRSA strain (ATCC 43300). Consequently, further investigations involving MSSA and MRSA clinical isolates, and polymicrobial biofilms should be conducted to determine whether the frequency-selective response observed here represents a general feature of bacterial biofilms or reflects strain-specific characteristics.

Moreover, physicochemical processes potentially involved in the interaction between alternating electric fields and bacterial biofilms, including reactive oxygen species generation, local pH changes, electrochemical reactions, membrane polarization, and electrical impedance variations, were not directly investigated. Likewise, although colony-forming unit enumeration and crystal violet staining provide complementary quantitative information on bacterial culturability and total biofilm biomass, they do not directly visualize structural alterations within the biofilm architecture. Therefore, the structural perturbation of the extracellular polymeric substance (EPS) matrix proposed in the present study should be regarded as a plausible interpretation of the observed frequency-selective antibiofilm response rather than as an experimentally demonstrated mechanism. Future studies integrating electrochemical characterization, electrical impedance spectroscopy, confocal laser scanning microscopy, scanning electron microscopy, and biochemical analyses of the EPS matrix will therefore be essential to directly correlate frequency-selective electrical stimulation with structural and physicochemical modifications of mature biofilms.

Finally, the study was conducted under in vitro conditions, and further investigations are needed to validate these findings in clinically relevant models.

## 5. Conclusions

The present study demonstrates that the antibiofilm activity of low-intensity electric fields is strongly frequency-dependent and is not uniformly distributed across the investigated spectrum. The results show that the antibiofilm activity is confined to a restricted frequency band (1–100 kHz), within which multiple frequencies are capable of reproducing the observed effect, indicating that the overall response is driven by specific effective components rather than by a cumulative spectral contribution. These outcomes support the concept of frequency-selective antibiofilm activity in *Staphylococcus aureus* biofilms and suggest that electric fields primarily act through biophysical mechanisms involving modulation of the cell membrane and extracellular matrix. The observed behavior is consistent with frequency-dependent responses characteristic of complex viscoelastic systems, thereby further reinforcing the hypothesis of a resonance-like interaction between the applied field and the biofilm structure. From a translational perspective, the identification of discrete effective frequencies within a broader spectral range creates the potential for the development of targeted electrical stimulation protocols that may achieve comparable or improved antibiofilm efficacy while reducing energy input and exposure time. Future studies should concentrate on refining the frequency-response profile, investigating multi-frequency interactions, and validating these findings in clinically relevant models.

## Figures and Tables

**Table 1:** Classification of electrical exposure protocols (EEPs) and investigated frequency ranges.

Protocol (EEP)	Frequency Condition	Frequency (Range or Value)
EEP1	FFR	10 Hz–10 MHz
EEP2	SR1	10 Hz–1 kHz
EEP3	SR2	1 kHz–100 kHz
EEP4	SR3	100 kHz–10 MHz
EEP5	SF1	1.62 kHz
EEP6	SF2	3.36 kHz
EEP7	SF3	8.86 kHz
EEP8	SF4	14.39 kHz
EEP9	SF5	61.6 kHz

**Table 2:** Colony-forming units (CFU/mL) in mature *Staphylococcus aureus* biofilms following electric field exposure across different frequency ranges.

Descriptive Statistics		
Group	*n*	Mean ± SD log_10_ (CFU/mL)
CT ^a^	3	8.56 ± 0.18
FFR ^b^	3	7.56 ± 0.05
SR1 ^a^	3	8.49 ± 0.13
SR2 ^b^	3	7.52 ± 0.11
SR3 ^a^	3	8.52 ± 0.08

One-way ANOVA: F(4, 10) = 28.77, *p* < 0.001. Values are expressed as mean ± SD. Different superscript letters indicate statistically significant differences between groups according to Tukey’s post hoc test (*p* < 0.05).

**Table 3:** Biofilm biomass quantification in mature *Staphylococcus aureus* biofilms following electric field exposure across different frequency ranges.

Descriptive Statistics		
Group	*n*	Residual Biomass (% of CT) Mean ± SD
FFR ^a^	3	41.64 ± 2.27
SR1 ^b^	3	91.10 ± 1.91
SR2 ^a^	3	40.43 ± 1.07
SR3 ^b^	3	88.03 ± 1.76

One-way ANOVA: F(3, 8) = 723.67, *p* < 0.001. Values are expressed as mean ± SD. Different superscript letters indicate statistically significant differences between groups according to Tukey’s post hoc test (*p* < 0.05).

## Data Availability

The original contributions presented in this study are included in the article. Further inquiries can be directed to the corresponding authors.

## Abbreviations

The following abbreviations are used in this manuscript:

EPS	Extracellular Polymeric Substance
PET	Polyethylene Terephthalate
RMS	Root-Mean-Square
MRSA	Methicillin-Resistant *Staphylococcus aureus*
MSSA	Methicillin-Susceptible *Staphylococcus aureus*
BHI	Brain–Heart Infusion
EEP	Electrical Exposure Procedure
EET	Electrical Exposure Time
PBS	Phosphate-Buffered Saline
FFR	Full Frequency Range
SR1	Sub-Range 1
SR2	Sub-Range 2
SR3	Sub-Range 3
SF1	Single-Frequency 1
SF2	Single-Frequency 2
SF3	Single-Frequency 3
SF4	Single-Frequency 4
SF5	Single-Frequency 5
DTT	Dithiothreitol
CFU	Colony-Forming Unit
CV	Crystal Violet
